# Correction: Brochet et al. A Quantitative Comparison between Shannon and Tsallis–Havrda–Charvat Entropies Applied to Cancer Outcome Prediction. *Entropy* 2022, *24*, 436

**DOI:** 10.3390/e24050685

**Published:** 2022-05-13

**Authors:** Thibaud Brochet, Jérôme Lapuyade-Lahorgue, Alexandre Huat, Sébastien Thureau, David Pasquier, Isabelle Gardin, Romain Modzelewski, David Gibon, Juliette Thariat, Vincent Grégoire, Pierre Vera, Su Ruan

**Affiliations:** 1 LITIS, Quantif, University of Rouen, 76000 Rouen, France; thibaud.brochet@univ-rouen.fr (T.B.); jerome.lapuyade-lahorgue@univ-rouen.fr (J.L.-L.); alexandre.huat@univ-rouen.fr (A.H.); sebastien.thureau@chb.unicancer.fr (S.T.); isabelle.gardin@chb.unicancer.fr (I.G.); romain.modzelewski@chb.unicancer.fr (R.M.); pierre.vera@chb.unicancer.fr (P.V.); 2 Centre Henri Becquerel, 76038 Rouen, France; 3 Société Aquilab, 59120 Lille, France; david.gibon@aquilab.com; 4 Département de Radiothérapie, Centre Oscar Lambret, 59000 Lille, France; d-pasquier@o-lambret.fr; 5 Département de Radiothérapie, CLCC Francois Baclesse, 14000 Caen, France; juliette.thariat@cfb.unicancer.fr; 6 Département de Radiothérapie, Centre Léon Berard, 69008 Lyon, France; vincent.gregoire@crcl.fr

## Addition of Authors

Alexandre Huat, Sébastien Thureau, David Pasquier, Isabelle Gardin, Romain Modzelewski, David Gibon, Juliette Thariat and Vincent Grégoire were not included as authors in the original publication [1]. The corrected Author Contributions Statement appears here. The authors apologize for any inconvenience caused and state that the scientific conclusions are unaffected. This correction was approved by the Academic Editor. The original publication has also been updated.
